# Mapping decision-making pathways: Determination of intervention entry points for diagnostic tests in suspected serious infection

**DOI:** 10.3310/nihropenres.13568.1

**Published:** 2024-06-07

**Authors:** Raasti Naseem, Nicola Howe, Sara Pretorius, Cameron Williams, Clare Lendrem, Philip Pallmann, Enitan D Carrol, PROTECT study team

**Affiliations:** 1 Health Innovation North East and North Cumbria, England, NE4 5PL, UK; 2 NIHR Newcastle In Vitro Diagnostics Co-operative, Newcastle University, Newcastle upon Tyne, England, NE2 4HH, UK; 3 Centre for Trials Research, Cardiff University, Cardiff, Wales, CF14 4YS, UK; 4 Institute of Infection, Veterinary and Ecological Sciences, University of Liverpool, Liverpool, England, L69 7BE, UK

**Keywords:** Antimicrobial resistance, care pathway analysis, platform trial, adaptive design, bacterial infection, implementation

## Abstract

**Background:**

PROTECT ( Platform Randomised evaluation of clinical Outcomes using novel TEChnologies to optimise antimicrobial Therapy) has brought together a team of researchers to design a platform trial to rapidly evaluate and adopt into care multiple diagnostic technologies, bringing immediate benefit to patients. Rapid diagnostic tests will be used to identify patients at risk of deterioration from severe infection, before they become critically unwell. The platform will assess their comparative clinical and cost-effectiveness relative to current standard of care. Preliminary work, conducted under a Health Technology Assessment Application Acceleration Award, provided key evidence to optimise the design of the PROTECT platform.

**Methods:**

Qualitative methods which involved consulting key stakeholders in the field of serious infection addressed the key priorities. A high-level care pathway analysis focusing on serious infection in secondary care, captured the points of contact, actions, decisions, and potential outcomes associated with a patient’s care.

**Results:**

Two use cases of rapid diagnostic tests for serious infection were identified; (1) in acute emergency medicine to decide on antimicrobial initiation and/or escalation of care, and (2) in hospitalised patients to monitor treatment response. Within the emergency department, clinicians believed a test would be used as an adjunct to existing standard of care and would be used to either rule-in or rule-out serious infection. The “ideal” test should be rapid, point-of-care, cheap to procure, have capacity for high usability, and ability to be performed and interpreted by all staff. Facilitators to the adoption of infection diagnostic tests is their clinical need, and the main potential barrier is poor change management and behavioural change.

**Conclusions:**

Any new test should provide robust evidence of its effectiveness and have the potential to benefit the clinical pathway for patients, clinicians, and hospitals as a whole, to be considered for adoption as a new standard of care.

## Plain Language Summary

Antibiotic resistance, where antibiotics no longer work against infections caused by bacteria, threatens the lives of millions of people globally. To prevent resistance, antibiotics should only be used when absolutely necessary. When patients present to emergency care with a suspected infection, it is difficult for healthcare professionals to know if it is caused by bacteria (which could need treating with antibiotics) or a virus (which cannot be treated with antibiotics), or something else. There is no reliable rapid test which can confirm bacterial infection, and current laboratory tests take too long to give results.

Newly developed diagnostic tests may help healthcare professionals make rapid decisions about whether to start antibiotics for severe infection, and to identify which patients need admission to hospital. These tests have not yet been thoroughly evaluated to see if they are useful in the NHS.

The PROTECT trial uses a platform design to evaluate several infection diagnostic tests rapidly to determine whether these improve care and patient outcomes, are safe, reduce the use of unnecessary antibiotics, and are value for money for the NHS. This research describes initial work carried out with experts in serious infection to find out which kinds of tests would be most useful to healthcare professionals in hospitals, and which type of tests should be included in the platform trial. We also explored with the experts what barriers there might be to introducing new tests into hospitals or emergency departments.

The PROTECT trial will have a diverse PPI group with lived experience of severe infection and/or lived experience of being part of a marginalised minority ethnic community. The PPI group have already influenced the study’s design by advising on the acceptability of new tests, the platform trial design, consent methods, communication of information and outcomes that matter to patients and their families.

## Introduction

Antibiotic resistance occurs when bacteria that cause infections become resistant to antibiotics. It represents a threat to the lives of millions of people around the world if urgent action is not taken. In 2022, approximately 58,224 people in England had an antibiotic resistant infection, an increase of 4% since 2021 ( https://www.gov.uk/government/news/antibiotic-resistant-infections-and-associated-deaths-increase). From 2021 to 2022, deaths associated with a severe antibiotic resistant infection also increased ^1^. To combat the growing problem of antibiotic resistance, the use of antibiotics should be limited to only those patients who absolutely need them. Approximately 20% of antibiotics are overprescribed in primary and secondary care in the NHS ^1, 2^, compromising the safety and wellbeing of current and future patients due to adverse effects of antibiotics and contributing to the ever-growing threat of antimicrobial resistance (AMR). Safely reducing patients’ exposure to unnecessary antimicrobials is therefore a national and global priority. Recent studies have focused on single biomarkers to optimise antibiotic decision-making in different patient sub-populations and at different points along the care pathway ^3– 7^ ( https://www.gov.uk/government/publications/addendum-to-the-uk-5-year-action-plan-for-antimicrobial-resistance-2019-to-2024). While these studies answer crucially important clinical questions, they are costly and slow to generate clinical impact. The UK 5-year action plan for tackling AMR, updated in May 2022, recommends: a) building the evidence base to support antimicrobial stewardship interventions, b) developing research methods for improving and supporting clinical confidence in diagnostic testing, and c) randomised trials to compare duration of antibiotics and examine impact on clinical and economic outcomes ^8^.

When patients present to emergency care with a suspected infection, it is often difficult for healthcare professionals to know if the infection is caused by bacteria (which could require antibiotics) or a virus (which may be treatable with anti-viral medications but cannot be treated with antibiotics). There is no reliable test which can rapidly confirm bacterial infection, and typically, laboratory tests take 24–48 hours to give results. This means that, often, antibiotics are prescribed to the patient before confirmation of the presence or type of infection.

Healthcare professionals worry about missing a diagnosis of sepsis, a common, potentially life-threatening complication of infection. The best treatment for sepsis includes early recognition, and prompt administration of antibiotics (for bacterial sepsis) and fluids delivered intravenously (IV). The unavailability of rapid, point-of-care tests for bacterial infection, and concern about delaying treatment for possible sepsis, has led to over-prescribing of antibiotics. There are new technologies which may help clinicians make decisions about whether to start, stop, or change antibiotics; however, their clinical and cost-effectiveness have not previously been evaluated in a large trial. A particularly efficient way of doing that is in a platform trial which allows multiple technologies to be assessed within the same trial, alone or in combination, wherever patients are seen with suspected infection. The Covid-19 pandemic showed that new treatments can quickly and safely be brought into clinical use by conducting platform trials, which allow faster decisions, compared to conventional two-arm trials, about which new treatments and tests should be used routinely to improve patient safety and care.

PROTECT was established to bring together a team of researchers to plan and prepare a platform trial, where multiple diagnostic technologies for suspected serious infection can be evaluated rapidly and, if shown to be safe and clinically and cost-effective, adopted quickly into care to benefit the patient. The team have constructed a flexible, adaptive platform trial design to comprehensively evaluate commercially available interventions across the patient pathway, in order to robustly establish clinical utility. The proposed platform, embedded in routine NHS care, is designed to address the complex problem of antimicrobial optimisation in a clinical area where diagnostics which support immediate clinical decision-making can enhance quality of care and patient safety, and reduce the risk of complications. Platform trials offer higher efficiency than individual trials of single biomarker interventions as tests can be evaluated concurrently (rather than sequentially), and the requirement for a separate control group for each comparison is removed. In a platform trial, an intervention can be eliminated from the trial where it does not demonstrate clinical benefit, and new interventions that become ready for evaluation can be added. Additionally, where there is evidence that an intervention is clinically beneficial, its evaluation can cease, and the intervention can be rapidly adopted into clinical practice. In this way, novel technologies can be introduced into the care pathway sooner ^9– 11^. PROTECT was awarded a Health Technology Assessment (HTA) Application Acceleration Award in 2022 to develop a master protocol and full HTA application. In November 2023, a proposal was submitted for a full trial to the research funder.

The PROTECT Acceleration Award encompassed mapping decision-making pathways for patients with suspected infections and determining intervention entry points in the clinical pathway. Information on care pathways for patients potentially at risk of deterioration from severe bacterial infection was elicited by the NIHR Newcastle In Vitro Diagnostics Co-operative (NMIC). The aims were to identify:

- Entry points along care pathways for those with suspected infection.
- Current practice in testing for serious infections across settings, including timepoints along the pathway where decisions are made regarding antibiotic treatment and criteria-based progression from pre-hospital to admission to ward.
- Points in the care pathway where interventions could be introduced for maximum clinical and cost-effectiveness and efficiency.
- Barriers and facilitators to test implementation.

These aims were addressed by carrying out qualitative research in the form of literature review, care pathway mapping and stakeholder group discussions, interviews and panel discussions.

## Methods

### Patient and public involvement

Patient and public involvement for the PROTECT trial began as part of the Accelerator award where significant efforts were made to involve the public in developing the PROTECT platform trial. We have three public involvement co-applicants and established an active diverse patient and public involvement (PPI) group of 30 members with lived experience of presenting unwell to emergency care and/or lived experience of being in a marginalised minority ethnic community. We have support from Sepsis Research FEAT, UK Sepsis Trust, ICU Steps and Antibiotic Research UK, who will connect us to wider PPI groups.

Three focus groups were conducted: one with children, young people and parents, and two with our adult PPI group. These groups helped shape the study design and outcome measures. They considered acceptability of new tests, perceptions of antibiotic therapy, risks of antibiotic resistance, methods of giving consent, the platform trial design and communication of research information in accessible multi-media formats.

Tackling AMR and evaluating new tests for severe infection were viewed as urgent problems. Both deferred and electronic consent methods were considered acceptable as long as clear justification was provided, and needs of diverse participant groups were considered (e.g. elderly populations or minority language speakers).

For the full PROTECT trial, the PPI forum, woven into all work packages and governance, will meet 2–3 times/year to advise on study information, recruitment, and selection of new tests, to improve participant experience. They will be supported by the PPI lead and the 3 PPI co-applicants, and will co-design all patient-facing documentation including the video information, consent and translation tool in multiple languages. Two members will join the Trial Management Group. We will use the Public Involvement in Research Impact Toolkit (PIRIT) as a checklist of PPI activities and relevant standards.

The PPI forum had no direct participation in the design or conduct of this preliminary work, however public involvement co-applicants attended and contributed to the stakeholder meetings in June and September 2023 and attended regular online meetings where the preliminary work was discussed.

### High-level care pathway analysis

To identify patient entry points along a care pathway for those with suspected serious infections, a high-level care pathway analysis (CPA) was conducted. CPA facilitates the identification and mapping of clinical events, actions, decisions and outcomes within the current pathway for a certain condition or conditions ^12^. The pathway elicited was an initial high-level outline, representing the heterogeneous range of infection types which people present with in secondary care that have the potential to develop into serious infection, rather than a detailed care pathway for a specific infection. The care pathway was mapped out using Lucid Chart, from an evaluation of literature and guidelines for serious infections.

### Stakeholder group discussions

A PROTECT stakeholder event was held in Leeds in June 2023 and included both core and wider project team members as well as select external participants. Small-group discussions, lasting one hour, were run with random placement of participants into one of five groups of 5–6 people. A discussion guide was drafted and followed. The aim of the discussions was to determine:

•  Which infection types in which population(s) are most common, severe, and difficult to diagnose?

•  What are the unmet needs in infection diagnostics?

•  What is the current pathway for infection diagnosis and where could a new test fit?

•  What are the implementation barriers and facilitators to test adoption?

**Figure 1.**
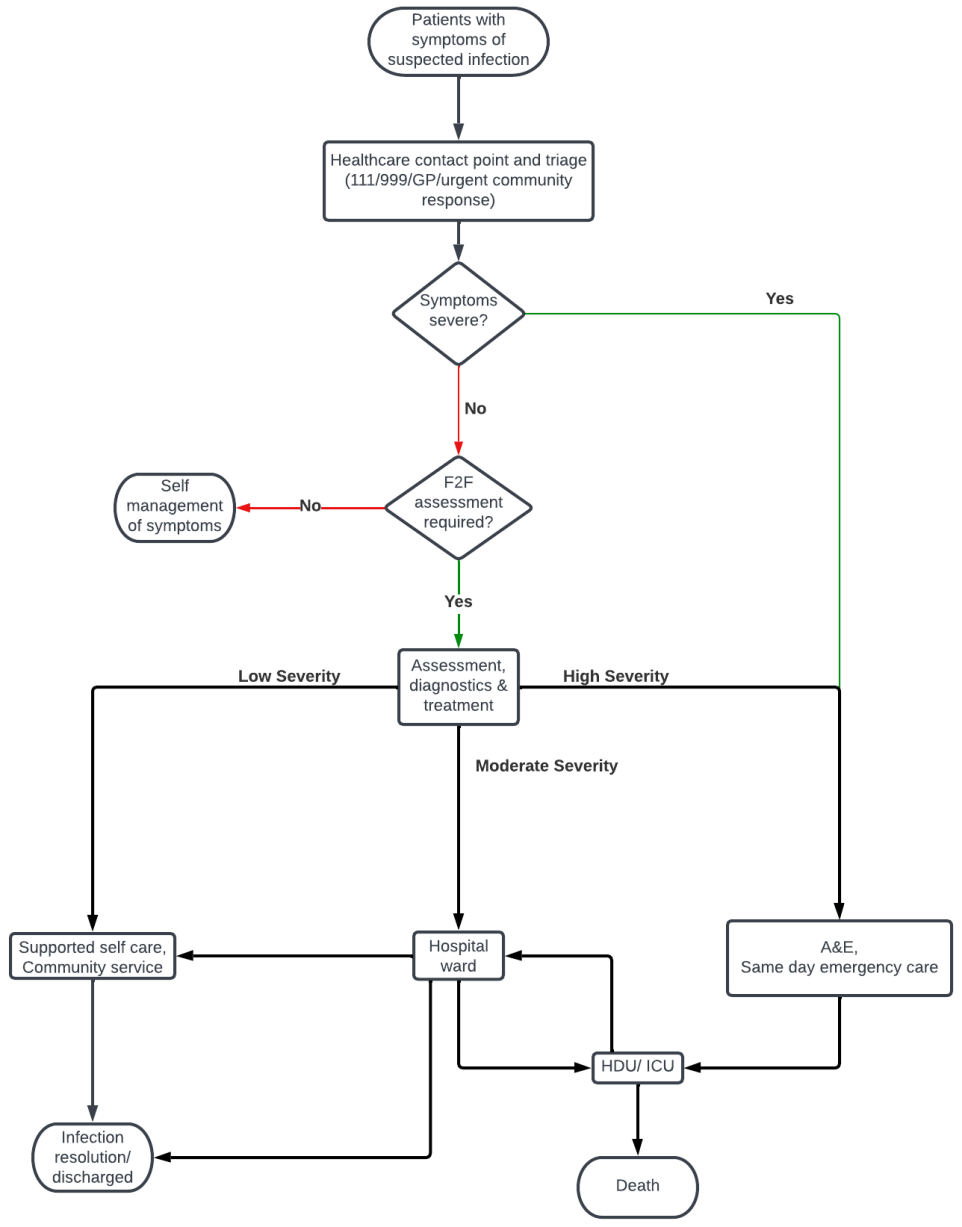
High-level care pathway map for patients with suspected infection.

To prompt discussions, stakeholders who participated in the five small-group discussions were provided with a high-level care pathway map ( Figure 1) and a set of prompt questions.

The main outcomes from the stakeholder discussion were to (i) inform the PROTECT project remit and (ii) inform the platform trial inclusion/exclusion criteria. The discussions also facilitated the development of questions for the follow-up semi-structured interviews. Data from these small-group discussions were summarised by two methodologists.

### Semi-structured qualitative interviews

The results from the small-group elicitations were further expanded upon in eight semi-structured interviews with additional clinical experts. Interview guides were developed to ensure the interviews addressed the main themes and aims of interest in developing the platform study. The guide was designed to elicit information on:

•  Current clinical management decisions for patients presenting with suspected severe infection.

•  New tests for infection, addressing AMR and supporting optimal antibiotic use:

- Where they would be best placed (setting)
- Where in the care pathway their use would be most appropriate
- Preferred test characteristics
- How the introduction of these tests would alter clinical decision-making.

•  Barriers and facilitators to test adoption (implementation).

Interviews were carried out from August to September 2023. At least two methodologists were present during each interview. Interviews lasted between 45 minutes and one hour and were conducted online, using Microsoft Teams. Interviews were recorded for note-taking purposes with all recordings consented to by participants. Quotes from analysis of the recordings are anonymised below. Interview data was analysed by two methodologists and key findings extracted.

### Ethical approval and consent

The study received ethical approval from the Newcastle University ethics committee on 25th April 2023 (Ref: 30913/2023). Written or verbal audio-recorded informed consent was obtained from all participants prior to interviews taking place.

A participant information sheet and consent form were provided to participants via email prior to the interview date and participants were asked to return the signed consent form prior to the interview taking place. Participants who did not return the signed consent form were asked to consent verbally during the video call. In these instances, and in accordance with Newcastle University recommendations for consent, one methodologist would read out the statements from the consent form to the participant and a second methodologist would witness and record the verbal consent on the consent form.

### Clinical expert discussion panel

A PROTECT stakeholder meeting for industry partners was held in London in September 2023. As part of this event, a clinical expert panel discussion was held with three emergency medicine clinicians with a discussion topic of “Expert perspectives on evaluating a diagnostic test in the Emergency Department.” From this discussion, key points were recorded and summarised by four methodologists.

## Results

### High-level care pathway analysis

A high-level care pathway map was produced based on reviews of relevant published literature and clinical guidelines for infections. Guidelines reviewed included the National Institute for Health and Care Excellence (NICE) guidelines for respiratory tract infections (RTIs) ( https://www.nice.org.uk/guidance/conditions-and-diseases/respiratory-conditions/respiratory-infections), urinary tract infections (UTIs) ( https://www.nice.org.uk/guidance/ng109, https://www.gov.uk/government/publications/urinary-tract-infection-diagnosis) and sepsis ( https://www.nice.org.uk/guidance/ng51). The pathway ( Figure 1) was verified by stakeholders. The purpose of the high-level pathway was to provide a broad overview of how patients move through a clinical secondary care setting with suspected infection, and to act as a prompt to further facilitate stakeholder discussions.

### Stakeholder group discussion

Forty stakeholders participated in the five small-group stakeholder discussions and provided feedback on the high-level care pathway and responded to guided discussions. Information gathered from participant sheets was not necessarily reflective of all who attended the discussions, as providing feedback individually was not compulsory. Responses gathered were anonymous.

When collating responses for the most common, severe and difficult to diagnose infection types, stakeholders were able to provide multiple, open text responses ( Table 1). The most commonly noted infection was RTI, followed closely by UTI.

**Table 1.** Most common infection types seen by participants in their clinical practice.

Infection Type	Responses (n = 49)
Respiratory tract	13
Urinary tract	12
Sepsis	9
Skin and soft tissue	8
Intra-abdominal	3
Bone and joint	3
Central nervous system (meningitis)	1

***Unmet needs*.** Two key priority areas for exploration in the small-group discussions were; (i) where the greatest clinical uncertainty for prescribing antibiotics lies and (ii) the current issues with the infection diagnostic care pathway. Group discussion elicited a broad range of responses. All five groups discussed the importance of being able to differentiate between viral and bacterial infections, and how this information can inform clinical decision-making. The unmet clinical needs which were identified included both diagnostic (differentiating between colonisation and infection, or between bacterial, viral and inflammatory) and a prognostic test (differentiating between severe and non-severe clinical status). An example of such a test would be one which could differentiate between colonisation and UTI. Another unmet need which was considered important was a test which could indicate bacteraemia in non-symptomatic patients. Tests that could inform decisions around hospital admissions and patient management were also highlighted as an unmet need. This included tests to support clinical decisions to send patients home (rule-out test), and to stop antibiotics or de-escalate from broad-spectrum to narrow-spectrum or IV to oral (to prevent overuse). There was enthusiasm for tests that would facilitate appropriate discharge of patients safely and how this would benefit the healthcare system. Test acceptability, non-invasive testing for children, and testing requirements (i.e., in terms of who would be trained to use the equipment and how much staffing time it would take to run a test from start to finish) were all raised as key considerations.

***Care pathway*.** As questions pertaining to the care pathway were not confined to a specific infection, discussions around the current care pathway were wide-ranging. Groups discussed how patients generally attend care settings with suspected serious infection, ranging from self-presentations (walk-ins) to transfers by ambulance. Participants felt that discussions regarding diagnostic testing for infection should be extended to testing where rule-out tests could avoid hospital admissions, which can be detrimental in elderly patients in care homes. It was highlighted that those presenting with infections will present differently, at varying levels of infection severity and through different routes. Clinical observations were noted as being fundamental to assessing a patient’s health status.

Participants identified that a new infection diagnostic test would be helpful in the clinical management of three main populations: (i) patients with non-specific symptoms, (ii) frail/elderly patients (where there is a high degree of clinical uncertainty) and (iii) the general population of those presenting unwell.

***Barriers and facilitators to implementation*.** Participants discussed barriers and facilitators to the adoption of a new test into clinical practice. There is a need for local and national validation of the tests that may be included in a study such as PROTECT, and clinical evidence for the test in a real-world setting to encourage adoption. In particular, it would be desirable to have an easy-to-read demonstration of clinical, economic, and wider benefits of the tests which are being trialled. Early stages of implementation should involve not only clinicians who would use the test but also a broad spectrum of stakeholders who are not patient-facing e.g., laboratory staff (pathology, biomedical scientists, biochemists), business managers etc. Involving these individuals in the adoption process early would ideally alleviate any resistance or challenges which might occur at a later stage of adoption. Additionally, local information technology (IT) support would be required in order to facilitate integration of the devices with patient records and local IT systems and reporting of test results in the electronic patient record (EPR).

***Platform trial remit*.** There were two key outputs driven by the information collected from the small stakeholder group discussions. These were the refinement of the project remit (i.e., goals and extent of the wider HTA project) and the platform trial inclusion/exclusion criteria (i.e., which settings and patient populations to include/not include in the trial).

It was agreed that the diagnostic tests used in the trial should:

1. Be able to provide clinically actionable information to support clinical decisions about antimicrobial optimisation in real time.
2. Be able to identify those patients at risk of deterioration from serious infection before they become critically unwell.

Table 2 presents the PROTECT platform trial inclusion/exclusion criteria for participants. The main inclusion criterion was refined to: people presenting acutely unwell with early signs and symptoms consistent with suspected serious bacterial infection, also referred to as suspected (bacterial) sepsis. This would be relevant for those patients presenting to a participating: emergency ambulance service, walk-in centre, out-of-hours/urgent treatment centre, virtual ward (“hospital at home”) or ED (to include but not limited to, ED observation unit, acute medical/surgical assessment unit, and same-day emergency care).

**Table 2.** PROTECT proposed inclusion/exclusion criteria for the platform trial.

Inclusion	Exclusion
Adults (≥16 years of age) with deferred consent, written consent or witnessed verbal consent/assent as appropriate by personal consultee or next of kin.	Patients already receiving IV antibiotics.
Children (<16 years of age) with deferred consent, written consent or witnessed verbal consent/assent given by parent/guardian, or assent by child if old enough.	Patients who are currently receiving myeloablative chemotherapy.
Presenting with signs and symptoms consistent with suspected serious bacterial infection (to include “suspected sepsis”).	Patients who have had solid-organ transplantation, allogeneic bone marrow or stem cell transplantation within three months prior to randomisation.

### Semi-structured qualitative interviews

Eight participants from five specialties including ED consultants (adult and paediatric), research paramedic, intensive care consultant and critical care nurse were interviewed. Participants were located across the UK in with experience in managing and treating patients with suspected serious infections ( Table 3).

**Table 3.** Interviewee participant information.

Identifier Code	Job Role
PRO_1	ED consultant
PRO_2	Paediatric ED consultant
PRO_3	Research paramedic
PRO_4	Intensive care and acute consultant
PRO_5	Paediatric ED consultant
PRO_6	ED consultant
PRO_7	Critical care nurse (and adult nursing lecturer)
PRO_8	ED consultant

The interview guide created for the semi-structured interviews covered three broad discussion areas: (i) current procedure for diagnosing infection, (ii) uses and characteristics of tests which could potentially be added into the care pathway of patients with suspected infection (including clinical scenario, clinical setting, patient population and position in the care pathway) and (iii) implementation barriers and facilitators to adoption for tests which might be introduced into the care pathway for patients with suspected infection. Quotes from interview participants have been anonymised and appear in bold text below.

**Figure 2.**
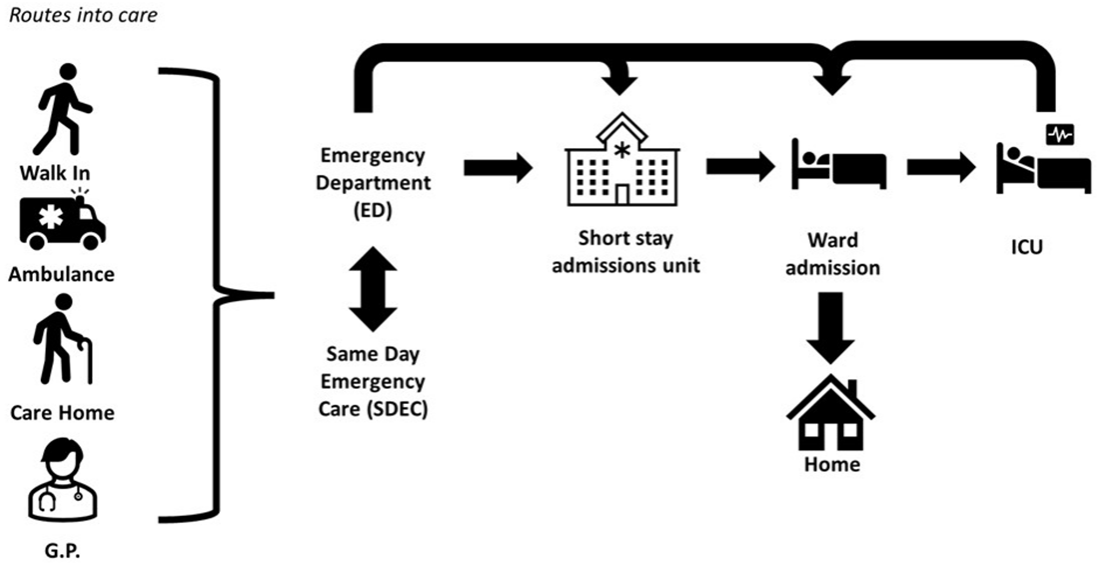
High-level overview of the care pathway for patients with suspected serious infection.

***Presentation of possible severe infection to a healthcare provider*.** Figure 2 presents a high-level overview, summarised from the perspective of the interviewees, of routes into care and the care pathway for patients with suspected serious infection entering the healthcare system. When interviewees were queried about routes into care, it was found that those patients at risk of severe infection present in a range of manners into the healthcare system. These include walk-ins to the ED, same-day emergency care, and via ambulance, NHS 111 (NHS 24 in Scotland), general practitioner (GP) and pharmacist referrals. Participants stated that, generally, the ED was the main hub for the admission of patients to secondary care. Optimisation of care via a septic system pathway in the ED or on a ward should prevent a patient’s admission to the intensive care unit (ICU).

Patients with suspected serious infection have varied presentations, with no one unique symptom indicating to clinicians that a patient has the potential to become seriously ill. Classic symptoms of infection include feeling unwell, feeling shivery and a high temperature. Other symptoms noted from participants included pain (e.g., abdominal, chest), shortness of breath, confusion, increased respiratory rate and increased heart rate.

**“Some people present with classical symptoms, but other people present with less obvious symptoms. It really can be quite a lot of different presentations, that's the challenge.” – PRO_6**

**“It can be lots and lots of different things.” – PRO_1**

Participants commented that they react to the symptoms observed, and diagnostic tests help to give a more complete picture of what is going on. Typically, a reasonable amount of clinical suspicion will lead to querying an infection in a sick patient. One clinician (PRO_5) commented, “what is the definition of being “sick enough”?” Interviewees stated that at present this evaluation would not be based on one decision at one point in time, but more likely a series of decisions and tools used to come to a "sick enough” conclusion.

Patients who present to secondary care will undergo a clinical assessment. Clinical assessment helps clinicians to stratify risk and to identify high-risk patients. Standard clinical observations for patients with possible serious infections typically include assessment of temperature, heart rate and respiration rate ( https://www.nice.org.uk/guidance/ng51). Clinicians may also confirm when the patient last passed urine and check the condition of the patient’s skin. There are some variations to standard clinical observations for very young or elderly patients, and observations may need to be adapted for community settings e.g., oxygen saturation may be difficult to measure in some settings. A general observation of the patient is also made, and clinical history is captured.

National Early Warning Score (NEWS) (now updated to NEWS2) or paediatric scores (PEWS) are early warning scores used to assess, monitor, and standardise care for patients either by GPs, by ambulance staff, or after presentation to acute secondary care ( https://www.rcplondon.ac.uk/projects/outputs/national-early-warning-score-news-2) ^13^. For NEWS2, a score is allocated for each of six physiological measurements taken during routine clinical observations, and then aggregated. These measurements are: temperature, respiration rate, oxygen saturation, systolic blood pressure, pulse rate and level of consciousness or new confusion. The higher the score for each measurement, the further it is from the normal range and the more frequent monitoring is required. Participants reported that a NEWS2 score of 3 in any single parameter (low to medium risk) would trigger frequent monitoring and prompt them to take blood samples for culture. A score of 5 should trigger immediate urgent review of the patient ( https://www.rcplondon.ac.uk/projects/outputs/national-early-warning-score-news-2). High NEWS scores (>7) or anything that indicates sepsis will lead to patients being “blue-lighted” to hospital with the hospital pre-alerted.

qSOFA (Quick Sequential Organ Failure Assessment, https://qsofa.org/) is a bedside prompt which may identify those patients with suspected severe infection at greater risk of a poor outcome outside of intensive care. It is an evaluation tool which uses three criteria: blood pressure, respiratory rate and altered mentation. A PROTECT team member indicated that qSOFA is used as an additional validated quick score system as well as the NEWS score, to evaluate those patients that require hospitalisation. As pre-hospital evaluators, it is difficult for paramedics to determine infection from clinical observations alone, therefore the qSOFA helps guide further intervention decisions.

The paramedic felt that, at times, use of the NEWS and qSOFA scoring systems led them to be over-cautious with patients “just in case” or because the score systems required it; thereby leading to an overdiagnosis and hospitalisation of patients who otherwise would have benefitted from primary or community care.

Patients with symptoms suggestive of a serious infection are likely to undergo investigative testing, depending on what clinicians suspect about the nature and location of the infection, where the patient presents (e.g., ED) and how their care is prioritised. This typically involves samples being assessed in a laboratory. The following tests were cited as being used, and are referred to in the NICE guidelines for assessment of suspected sepsis ( https://www.nice.org.uk/guidance/ng51):

•   A full blood count or basic bloods (haematology and biochemistry) is conducted in most patients, particularly in those with NEWS score indicating sepsis.

•   Blood gas including glucose and lactate measurements.

•   Chest X-ray for possible chest infections

•   Urine tests – presence of leukocytes and cytokines – for UTIs

•   C-reactive protein (CRP)

•   White blood cell count

•   Urea and electrolytes

•   Creatinine

•   Lumbar puncture for infants if meningitis is suspected

•   CT scan

Participants referred to existing point-of-care (POC) tests in the ED such as respiratory panel testing for influenza, SARS-CoV-2 and respiratory syncytial virus (RSV). Blood gases and POC troponin testing were also mentioned. Some participants also reported that they had access to POC blood testing in the ED for patients with suspected infections.

**“Any treatment that would be internationally recognised as falling within sepsis infection guidelines.” – PRO_8**

Treatment decisions are determined by using a combination of the scoring systems described above, clinical guidance (e.g., NICE guidelines or Sepsis Six manual: https://sepsistrust.org/professional-resources/clinical-tools/), clinical judgement and how sick the patient appears upon presentation. It was noted, however, that very few guidance documents quantify how sick someone should be in order to be treated. Commonly cited initial treatments for patients suspected of a potentially serious infection were the administration of IV fluids and oxygen, as indicated by NICE ( https://www.nice.org.uk/guidance/ng51). Participants noted they adhered to the NICE guidance for treatment of suspected sepsis ( https://www.nice.org.uk/guidance/ng51) and usually a broad-spectrum antibiotic would be administered whilst waiting for the results of tests in patients who had a suspected serious bacterial infection. In accordance with the NICE sepsis guidelines from 2016, relevant at the time of this research, these would be administered within one hour of the patient being seen. A small number of children of all ages would be treated with antibiotics on arrival if considered unwell enough. However, for those where there is less concern, clinicians can allow up to 3 hours for completion of investigations in order to make an informed clinical judgement about antibiotics. Participants stated that, in line with most international guidelines, treatment should be administered within three hours for the treatment of sepsis. Therefore, tests would also need to be administered and return results within this timeframe.

Interviewees also referred to the ‘Sepsis Six’. The Sepsis Six care bundle has been adopted by hospitals for the management of patients with sepsis, with the aim of increasing survival when all elements of the bundle are achieved ^14^. Based on the outcome of a scoring system (typically NEWS2, PEWS or modifications of these (MEWS)), the Sepsis Six care bundle is triggered. The Sepsis Six consists of a set of six tasks (involving tests and treatments) including oxygen, cultures, antibiotics, fluids, lactate measurement and urine output monitoring. IV antibiotics are to be administered according to local protocols. The care bundle is to be implemented within one hour by non-specialist practitioners at the frontline ( https://www.nice.org.uk/guidance/ng51). Participants noted that Sepsis Six testing, and triggers to perform it, are well known by staff.

Participants stated that a MicroGuide is referred to for antibiotic treatment. Empirical treatments are determined by the local microbiology department based on local microbiology epidemiology. Antibiotics would generally be issued before test results are returned in those patients where infection is suspected unless a rapid or instant POC test such as a urine dipstick test is used in which case antibiotic administration may follow the test result. This illustrates that current practice is not in line with current messages regarding AMR, where appropriate use of antibiotics should be built into healthcare delivery to help combat AMR. In terms of antibiotic stewardship, clinicians evinced anxiety about delaying antimicrobial treatment, because of the recommendation to treat within the first hour, concerns over the repercussions of not treating, and a perceived need to “do something” for the patient.

**“It's easier to do something, rather than not do something and then get criticised for not doing it. People want to feel like they're doing something for the patient.” – PRO_8**

Participants were cognisant of AMR and local guidelines (MicroGuide) indicating which antibiotics should be given (ideally not too broad-spectrum) but most did not necessarily consider AMR when deciding on treatment. Instead, some participants reported a conflict between antimicrobial stewardship and urgent patient care with the imminent risk to the patient of not treating with antibiotics perceived as greater than that of AMR. Participants thought that this perceived risk drove a lot of antibiotic prescribing in emergency situations.

**“Wherever there's a real risk of harm [to the patient] antimicrobial stewardship is very low down on the list of priorities.” – PRO_5**

**“I don't think it's thought about explicitly in an individual patient-by-patient basis in hospital.” – PRO_8**

Delaying or withholding treatment decisions with a view to antimicrobial stewardship was considered more harmful, if these treatments were then later found to be required, than prescribing antibiotics in circumstances where they were then not warranted. In other words, apprehension over patient safety always trumped concerns over AMR.

On the use of broad-spectrum antibiotics, participants explained that when presented with a patient with a possible serious infection where the infection type was as yet unknown, they needed to strike a balance between using broad-spectrum antibiotics to provide protection from the most severe infections and being too prescriptive in terms of tackling AMR. Waiting an indeterminate amount of time for the results of a test and prescribing a narrower-spectrum antibiotic was not considered to be realistic or beneficial to the patient in ED. Treatment decisions were also influenced by pressure from patients or patients’ relatives to administer antibiotics in the presence of potential infection. Participants felt that if this led to antimicrobial prescribing, they would have the opportunity to modify treatment later when test results were reported.

**“We tend to follow more empirical guidance and then later those antibiotics may be further justified, but that tends to be 24, 48 hours down the line.” – PRO_1**

In vulnerable patient groups there are special considerations for treatment; for example, in infants under three months of age, antibiotics are typically prescribed immediately for those with possible serious infection. For adults, it is dependent on how ill they appear to be. At one end of the spectrum, there are the patients who are clearly unwell and have been taken immediately or transferred to the resuscitation area (resus), who will get early empirical antibiotics before the source of infection is known. Others will get further tests to try and determine the nature of the infection but also empirical antibiotics. For other patients who are not so ill, practice varies: some will be prescribed antibiotics whilst others will need to wait for the results of blood tests before treatment. This indicates that handling of each case of possible severe infection varies greatly, and treatment decisions are based on patients’ clinical status as well as practitioners’ experience.

***Uses and characteristics of new tests for infection introduced into the care pathway*.** Interviews addressed where a new diagnostic test would have the most impact in the care pathway for patients at risk of serious bacterial infection. Interviewees were also asked to consider where their placement would be of most benefit to patients or to the reduction in AMR. The majority believed that the earlier in the pathway a test was placed, the greater the opportunity to affect patient and potentially AMR outcomes. Ideally a test would differentiate quickly between patients who have the potential to become seriously unwell from those whose condition is unlikely to deteriorate and who could possibly be discharged from a secondary care setting. Distinguishing between these two patient groups would occur ideally before treatment had been administered. If antibiotics had already been given, a new test would be less effective in tackling AMR. Participants reported that there were currently no effective tools to rule patients in or out in this way for serious infection.

The following scenarios were suggested by participants in terms of where a test to diagnose suspected infection early would be most beneficial, and the type of test which would be most fitting.

- *Use case 1: Infection diagnosis in acute emergency medicine*.

Within secondary care, acute medicine would be an ideal setting for a new test for diagnosing potentially serious bacterial infections to be introduced. Placed here, it would expedite the flow of patients to the appropriate settings (admittance or discharge) and alleviate some of the pressures that are presently imposed on EDs across the country. Interviewees discussed that an algorithm-guided test, for example prompted by reaching a set NEWS2/PEWS scoring threshold, could be enacted by staff, in triage, to flag patients who may benefit from early antibiotics in a four-hour window. The test would ideally fit in with current practice (before, after, or alongside other tests carried out). A test in this setting would need to be quick, as the rapidity of the triage process does not allow for in-depth assessment. In the ED, clinicians were generally more concerned with risk stratification (e.g., low/moderate/high risk) as opposed to infection diagnosis (i.e., what the infection was). It was noted that the “moderate risk” group were the patients who were the most difficult to diagnose and treat.

A definitive rule-in or rule-out test would help with the quick identification of patients who needed to be treated for potentially serious infections, and those who could be discharged or who could wait longer for treatment. The test would be an add-on test to those that are currently performed in the ED. Patients categorised as likely to become seriously unwell could undergo intensive monitoring or be escalated to ICU if they were critically unwell or required organ support. Patients with infections such as pyelonephritis that are not systemically unwell, have the potential to be safely discharged with appropriate treatment and remotely monitored using virtual wards once quickly diagnosed. A rule-out test was deemed more desirable in settings such as ED and triage over a rule-in test. In an ED setting, the key point is to obtain clinical confidence in whether patients can be safely sent home. This test type would help indicate which patients can be discharged without further treatment or investigations, or with appropriate treatment and followed up in the community.

**“I’d like it to say, with 99 plus percent certainty, this patient is safe to discharge.” – PRO_6**

**“If you could separate out the patients who could wait versus the ones who need to have treatment right now, it [the test] is worth its weight in gold.” – PRO_3**

**“[Rule-out] is the one that probably has the biggest impact, by allowing you not to treat lots of people with antibiotics [who don’t need them].” – PRO_6**

**“A test to make it more comfortable sending people home. In terms of prognosis, knowing that likelihood of deterioration post discharge would be important.” – PRO_8**

There were conflicting views about how early in the pathway a rule-out test should be positioned. On presentation to the ED, it was felt that it would be helpful to rule out the potential for serious infection in patients to facilitate patient management. However, this would risk ‘ruling out’ patients who do not initially score highly on screening tools and are therefore not subjected to follow-up testing, or those who are not showing the right ‘sick’ symptoms early but who could later go on to become very unwell. It was suggested that a new test would be best placed during or after assessment, i.e., once the clinician had a clearer picture of the patient’s clinical status.

**“There's a risk when you front load tests that you do too many and you do the wrong one.” – PRO_8**

For a test which could be used early in presentation, it was felt that there would still need to be a criterion or checklist with a dichotomous result (yes/no) that patients must meet in order for the new test to be subsequently deployed. An early rule-out test might also allow for quicker, safe discharge from the ED and a reduction in referrals to other departments. Additionally, the identification of infection early on in the triage process might allow for expedited treatment, thereby potentially preventing patient deterioration. This might lead not only to improved patient outcomes but also present an economic benefit to the NHS by freeing up beds and eliminating the need for further tests, treatment, and other resources.

The benefits of an effective rule-in test were also expressed, allowing the identification of patients at risk of becoming seriously unwell. It was considered that this type of test might hold particular benefit for patients in the so called “grey area” or “amber area”. These are patients who are showing no urgent signs and symptoms of serious infection and for whom it is unclear whether they are, or may become, seriously ill. Identifying these patients, as well as those rare patients with septic shock is a “needle in a haystack situation”. A rule-in test in the ED might provide additional granularity around a patient’s current severity of illness over and above standard tests to help identify which patients can be admitted non-urgently for further tests and investigations, and those to be escalated to an appropriate department if deemed high risk (i.e., high dependency unit (HDU) or ICU).

Participants talked about patients who should be treated in specific timeframes with regards to severity of infection or symptoms. Timeframes noted were treatment within 30 minutes, those who could wait an hour, and those who could wait three or four hours. A rule-in test to stratify these patients and categorise them in terms of risk would facilitate accelerated patient care for those who most urgently require it.

**“I want to know, is that patient going to deteriorate over the next few hours due to their serious bacterial infection? I want a test that's going to tell me that so I can give them early antibiotics, I can plan where that patient's going to go, I can plan how aggressive my fluid therapy is going to be to treat their shock. And overall, how aggressive my management’s going to be.” – PRO_1**

Participants described how time is of the essence for those critically ill patients with serious bacterial infection. Patients ill enough to require immediate admission to ICU are more likely than other patients to die within the first 24–48 hours. Therefore, it would be beneficial if a new test had a rapid result turn-around time in comparison to standard tests for diagnosing infection (e.g., urine and sputum microbiological culture, blood cultures). A test such as this would facilitate quick clinical decision-making in accordance with infection status. If the narrowest-spectrum antibiotic could be administered immediately this could be very beneficial for patient outcomes, rather than waiting 48 hours for results of culture, only to possibly find out that not only was the patient not improving but they had not been on the most effective treatment.

Implementing a new test concurrently with the current standard-of-care tests for infection diagnostics was thought to be the best approach by participants: ideally any new add-on test that would fit in well with current practice. It would also allow for standard-of-care testing to be carried out providing a holistic overview of the patient’s health in a manner that would not greatly disrupt the current care pathway. It was thought that to administer the new test after standard tests would be too late as, by then, infection is already suspected, and the patient is on a care pathway where treatment may have already started.

**“This test is likely to be part of the puzzle, isn't it like all of medicine, history, exam, it's all one more puzzle piece. Having that additional one, which performs well, would be useful.” – PRO_2**

The majority of participants believed that, of the patients presenting to the ED, those who would benefit most from a serious infection diagnostic test were the “indeterminate, amber risk” patients, i.e., those patients who are not critically unwell, and for whom there is some suspicion of severe bacterial infection, but the source is unclear and who may be sent home. This population are at risk of becoming seriously unwell and deteriorating quickly when they do.

**“It's those ones in the middle, ones [patients] really, hovering between referring them to elsewhere else, that generally always happens out of hours.” – PRO_3**

A priority population for testing would be those who are most vulnerable to infection. For example, patients who are neutropenic, immunocompromised, elderly, or very young children/babies.

- *Use case 2: Secondary care – monitoring treatment response.*

Participants expressed that a diagnostic test, to assess host response (biomarkers) and/or pathogen identification using molecular testing, could have an alternative use as a monitoring test, to assess treatment response in patients. This would most likely sit on a hospital ward or in the laboratory, due to the amount of time it can take for an antibiotic treatment to take effect (stated as being typically 24–48 hours).

**“It would be a useful guide to see if the patient is picking up.” – PRO_7**

**“It would let me know that I'm doing the right thing and they're not deteriorating. If they were deteriorating, and the infection was getting worse, it would make me think that the antibiotics aren't working, the infection is not in the right place.” – PRO_4**

A monitoring test would provide healthcare teams with confidence in what they are doing, in patients who had already been started on antibiotics. If there was no clinical response to treatment, or no improvement in test parameters, it would encourage further investigations and more aggressive treatment or escalation (for example referral to ICU) at an earlier timepoint rather than waiting and watching for the patient to recover. There might also be a benefit in terms of discharging patients from hospital quicker: if the test indicates a response to treatment and associated clinical response, they could be sent home sooner, without waiting for standard-of-care test results. A subgroup of patients in which a monitoring test might be useful are those for whom further assessment or treatment is needed but immediate antibiotic administration is not warranted. The ideal scenario would be that a patient’s symptoms would resolve from other therapies alone, without the use of antibiotics. One clinician commented that care is very expensive for this group, and this is where monitoring infection will be most advantageous.

- “ *Ideal” diagnostic test for severe infection: test specifications*

The following test characteristics ( Table 4– Table 6) were highlighted as important for a new test being introduced to the care pathway for patients with potentially severe infection. The interviews revealed that the required specifications of a test will vary based on where the test sits, its intended use (i.e., rule-in, rule-out, versus monitoring) and the patient population in which the test will be used. Any new test should also link directly to EPRs.

**Table 4.** Performance characteristic considerations for a diagnostic test.

Performance characteristic	
Sensitivity/specificity	For a highly efficient rule-out test, sensitivity is the prime consideration, in comparison to a rule-in test, where good test specificity is key. High test specificity could bring the greatest benefit to patients and is critical in indicating the presence of serious infection. **“A test that is around 80% accurate to rule-in, that would be very beneficial in my practice. What I don't want is a non-specific test that everyone has as that just means that there's no change to current practice whatsoever”. – PRO_1**
Accuracy	Participants discussed that a new test should be at least as accurate as any risk-based scoring system used currently in screening or triaging patients in the ED. It was recognised that no test was perfect, and some patients would always “slip through the net”; however, tests would be used in conjunction with clinical judgement and therefore seen as a decision aid. **“To get healthcare providers to change their behaviour, accuracy is needed.” – PRO_7**
Diagnostic ability of test – infection	Participants stated that it would be useful to have a test that indicates (ideally yes/no) with a high degree of certainty whether a patient has an infection and will deteriorate and become seriously ill. **“In terms of treating patients, you just need infection; yes, no.” – PRO_6** The threshold for serious infection levels must be carefully considered so as not to over- or underdiagnose patients. Where the patient is in the care pathway influences whether the test needs to identify the pathogen. Earlier on in the pathway (e.g., in the ED), a clinician needs to know whether the patient has a serious infection or has the potential to become seriously ill (recognition), then whether the illness will escalate whilst the patient is being treated (escalation). Later on, in order to de-escalate treatment, it would be useful to know what infection the patient has, if infection is present. This could be confirmed later with currently used testing procedures such as sending bloods for culture. The following capabilities were also noted as helpful in a test to assess serious infection: - identification of the bacteria causing the serious infection - distinguishing bacterial from viral infection - identifying infection versus colonisation.
Diagnostic ability of test – AMR testing	Although participants were aware that it may be unrealistic in a rapid test, some cited a test which assessed AMR as an unmet need. A test which could both identify infection species and perform antibiotic sensitivity testing would let clinicians know the cause of the infection and how they could treat the patient with a narrow-spectrum antibiotic as early as possible. **“I want to know what bug it is because obviously that affects your treatment choice. You can rationalise your antibiotics early, so having that as early in the pathway as possible, is preferable to me. I find it amazing that we just use empirical antibiotics, when we have the technology to actually know what we're treating and to optimise our treatment as early as possible.” – PRO_4**

**Table 5.** Operational characteristic considerations for a diagnostic test.

Operational characteristic	
Test type	Most participants believed that any test introduced to the ED should be POC, as close to the patient as possible. There were specific stipulations noted for a POC test to sit in the ED, the main one being that the test would be compact enough to fit in a busy and crowded ED. The test should also be robust, usable and accessible by all staff. **“It needs to be simple to do. It needs to be quick. The kits that it needs need to be cheap…the kit needs to be robust.” – PRO_8**
Sample type	The most discussed sample type amongst participants was use of a blood specimen. Participants who worked in acute care were more likely to suggest a finger prick test as a ‘non-invasive’ method of testing. Those who worked in ICU suggested that, as patients would likely have a venous line, and finger pricking could introduce another potential source of infection, a sample could be drawn directly from the line. Paediatricians highlighted that what counts as ‘non-invasive’ was entirely subjective and that small children could find venous blood tests stressful (they often end in failure and repeated attempts) and so a finger prick test would be most tolerated in this group.
Time to result	A test characteristic considered to be one of the most important was time to result. **“It probably matters less whether it's point-of-care or lab-based as long as turnaround is quick.” – PRO_8** A new POC test should be quicker to perform than existing tests, such as CRP, which are carried out in labs to assess patients for infection. This is especially important in an ED or where very sick patients are at risk of rapid deterioration. Speed can also reduce the chance of prescribing inappropriate antibiotics. Speed of results was also discussed as possibly being important for infection control procedures in the department, depending on the infection type.
Result analysis/output	A dichotomous result for infection presence was noted as being the ideal output for test results, giving a clear ‘yes/no’ indication of infection presence. Participants explained that a scale, numeric or ‘traffic light’ system would still need some interpretation and would be less useful. It was considered that choice of antibiotic was unlikely to change depending upon level of certainty regarding the presence of a particular microbial species, further supporting the use of a test which provides a binary (yes/no) response.
Ease of use	Ease of use was very important to participants. Tests should be intuitive and easy to use for all grades of staff, with simple instructions which are easy to memorise without staff having to refer to instructions with each use or after a period of absence. It was noted that reading instructions in a busy ED was impractical. Participants stated that they would prefer a test that is small and/or portable. Devices that are similar to smartphones or which implement touchscreens were popular. **“The test has got to be seen as being helpful, useful, and easy straight from the beginning because otherwise people just go off it straightaway.” – PRO_3**
Robust	Participants stated that the test and associated kit needed to be robust and fit for the environment. In a busy ED, for example, it would be required to withstand a high rate of usage. **“It's going to be able to cope with 150 samples a day done by 50 different people, which is not the same as 150 samples in a highly trained, controlled environment (a laboratory).” – PRO_8**
Training	The introduction of new tests requires time and staff training, ideally by the test developer. The test’s ease of use will likely determine the success of the training. As well as practical hands-on training, staff may be required to interpret results and understand the implications for patient care. For example, explaining the benefits in terms of number or percentage of patients expected to be positively affected. It would also be beneficial to understand how the new test ties in with current standard-of-care tests, and how these sets of results align.

**Table 6.** Economic characteristic considerations for a diagnostic test.

Economic consideration	
Test cost	Participants spoke about the cost of new tests, test consumables and maintenance. Even tests that are cheap to purchase and easy to perform can incur high costs if a lot of staff time is required to perform them or if they are used on a large population of patients. Alternatively, should a test be expensive to run, there would have to be some selectivity in terms of which patients the test was used for. New tests should be proven to be at least cost-neutral and ideally cost-saving to the NHS. If the test was to be used as an add-on (rule-in/rule-out), the cost would be saved through either sending the right patients home (rule-out) or sending patients home with appropriate treatment thus avoiding putting them through unnecessary additional testing and/or hospital admittance (rule-in). Participants suggested that a cost-benefit analysis should be performed for a new test to demonstrate potential cost saving and facilitate acceptance into practice.

***Diagnostic test implementation: barriers and facilitators*.** This study attempted to identify the barriers and facilitators to test implementation. Through identification of the barriers to implementation, an understanding of them can be developed and methods of combatting them can be devised. Studying implementation allows a better understanding of the ways in which research findings can be successfully promoted into adoption, adaptation, integration, scale-up and sustainability of evidence-based interventions ^15^.

Participants were asked what they considered to be the barriers and facilitators for implementing a new diagnostic test into the serious infection care pathway.

**Clinical need:** In order for a new test to be adopted it should meet at least some of the unmet clinical needs highlighted within this article. It should be a rapid and clear test which could evaluate “grey area” patients and those at highest risk of not receiving urgent care under current standard-of-care regimes due to not presenting typically as a patient with severe infection. It would provide shortened time to results relative to lab-based standard-of-care diagnostic tests. The test would facilitate patient flow within the ED and move people to appropriate routes of the care pathway in a timely manner thus easing pressure on the ED.

**Change management**: A challenge for the adoption of a new test is change management and ensuring that the staff affected are ‘on board’. A new test in any department could be seen as more work to do, and one participant explained that performing a new test might be perceived as “scope creep”, i.e., that the new test would be an additional task and would be more time consuming than current practice. This might be more likely in EDs where, as a resource-limited, overwhelmed department, a new test could be burdensome. Participants communicated that healthcare staff would need to be persuaded that a new test was as easy to use as other tests, that results are diagnostically valid, and that they would have a positive impact upon the patient, the care pathway and clinical team, without being a burden on their already heavy workload.

**“A lot of people have worked in these areas [EDs] a long time and find change quite challenging. Change to them can be seen as another thing added to their job. I think it has to be made very clear how it's going to make their job easier, rather than just another thing that they have to do each shift.” – PRO_7**

**Behaviour change:** Changing clinicians’ prescribing behaviour is notoriously difficult and depends on a number of contextual factors including experience, urgency, hierarchy, uncertainty and status/position within the larger hospital system. Clinician education focused on appropriate antibiotic use, antibiotic guidelines and prescriber feedback may improve antibiotic prescribing. Clinicians’ capability and motivation are potential drivers for changing prescribing behaviour.

It was suggested that to limit the extra workload created by additional tests, they should only be performed on select patient groups, not least because performing a test on all patients would be a waste of resources. One way to implement this would be to integrate the trigger for the new test with a clinical scoring tool such as NEWS/PEWS.

As part of the implementation plan, it would need to be clear how the test would be used, who would run the tests and who would be responsible for the test equipment and kits. For instance, would all healthcare staff interacting with patients in EDs be able to use it, or would it be solely nursing staff and doctors? Would a nurse or healthcare assistant be able to interpret results and act upon them, or would they need to wait for a doctor to interpret results or authorise subsequent actions? Would the laboratory be responsible for kit maintenance if it was a POC test in the ED, or would this fall on the ED staff? The introduction of a new test would likely place more pressure on some professionals whilst removing responsibility from others. Therefore, roles and responsibilities would need to be clearly defined.

A layer of resistance or ‘bureaucracy’ was described as coming from some laboratory or microbiology staff regarding the validity and verification of results. Potential gatekeepers of new tests were identified as microbiology or biochemistry departments.

**“Lab staff have done this [diagnostic testing] for years using the same techniques and they're so aligned to that. There's a resistance to change.” – PRO_4**

Some participants described resistance in the context of the conflict between the experience and clinical judgement of clinicians and test results which might not support their decisions. Clinical judgement can often trump the results of new tests.

**“You need to think strongly about how the clinicians are going to accept and act on the results. In some ways that’s more important than the accuracy of the test: whether the clinicians are willing to accept a new test.” – PRO_3**

Participants drew the distinction between those who were more conservative and sceptical, and those who were “early adopters”. The best way to convert those who were conservative was thought to be by providing consistent clinical and economic evidence. Data should also prove that a test works in more than just one geographical or hospital location and in a range of patient populations. It was recognised that, to initiate changes, there must be perseverance and that these things “take time”.

**“Persevering, presenting evidence, showing people that the test works. After a while, the culture starts to change, and then everyone just accepts that as part of standard care.” – PRO_4**

It can often begin with one NHS trust and healthcare team using a piece of validated test kit to spread the word through their contacts to get others on board.

**“What often is more effective is where you hear about someone doing an initiative somewhere, see that it works, then it kind of transfers more organically from one place to another.” – PRO_3.**

To affect change on a practical level, key stakeholders other than frontline healthcare staff including business managers, laboratory managers, commissioners, and procurement should be engaged.

**Outcome measures:** When evaluating the impact of a new diagnostic test for infection on the care pathway it is necessary to consider how introduction of the test might impact the patient (i.e., patient outcomes), healthcare providers (i.e., clinical team) and the healthcare system (i.e., NHS). Providing evidence of benefit to each of these stakeholders would facilitate test adoption.

For a rule-out test, which indicates whether a patient can be safely discharged, evidence of increased discharge rates, reduced admissions or reduced hospital bed days would support adoption. Further facilitatory evidence would include improved patient outcomes following a faster diagnosis, prescribing of the most appropriate antibiotics and an attenuation in the rate of AMR development.

Greater clarity in terms of the care pathway for those patients determined to be “amber” or “indeterminate” risk would be advantageous. Clinical management for patients at the extreme ends of the risk spectrum is more defined – low-risk patients can be discharged, and high-risk patients are often easily identified and treated. There is more uncertainty around moderate or unclear risk patients due to ambiguity around their presenting symptoms and test results.

Concern was expressed that it might be difficult to demonstrate the beneficial impact of a new diagnostic test on the health system despite evidence of clinical benefits to the patient. One ED consultant cautioned that there are other resource factors that control how long a patient may spend in the pathway or ED specifically, such as the time waiting to see a doctor or the number of other patients who are waiting to be seen by medics.

**“It's very difficult to maximise tests to optimise system performance when you've got ambulances queuing outside for 12 hours.” – PRO_1**

This was noted as a wider healthcare system issue and a problem due to current constraints on the NHS and EDs.

**Staffing requirements**: In order to enable the introduction of a new diagnostic test for infection, the staffing and functional requirements for running and reading the tests must be reduced compared with those for conventionally used tests. The requirement for less staff resource would alleviate some of the burden on busy medical departments. Included in this is also test usability across a broad spectrum of staff and the requirement for low maintenance training and upkeep of this.

**Test cost:** Cost was highlighted as a key factor influencing test adoption. A cheaper test than current standard of care would be more likely to be adopted. However, the cost would have to be weighed against the benefits and for a cheaper test there would need to be robust evidence of clinical benefit.

High costs for the test could be a barrier to implementation as the standard methods of testing for serious infection (e.g., blood tests and biochemical tests) are typically cheap to run and already well established in hospital laboratories. Furthermore, the introduction of a test which was expensive relative to standard of care (e.g., £300 versus £1 per test), even if clinical benefit was evidenced, might lead to selectivity in terms of who would receive the test.

Robust evidence of cost-effectiveness for the health service would be required to support its adoption. However, the requirement for an initial investment in the test, test kit, relevant infrastructure and training might overshadow such evidence.

**“If I had to go to my medical director and finance director and say we need £2 million up front to put in this new test that will save you, in three years’ time, £5 million they'll say ‘no’. They haven't got the money to be able to pay the two million, even if it's going to save over the next three years. It's really complicated.” – PRO_8**

**Performance requirement:** An additional suggested facilitator was evidence of ease-of-use. A simple single-step test would be easier to implement than a complex multi-step test which required a number of pre-test steps and complex analysis of results. A further suggested facilitator was evidence that the test was fit for the environment (e.g., robust in a busy ED where equipment might be subject to damage and frequent use).

### Clinical expert discussion panel

A panel discussion was held with three emergency medicine clinicians during a PROTECT industry stakeholder event in September 2023 in London. The topic of “Expert perspectives on evaluating a diagnostic test in the Emergency Department” was discussed amongst the panel and stakeholders present at the event. The key points arising from the panel discussion were extracted for consideration. The panel discussed the ideal requirements of a new test for the ED, with specific focus on antibiotic stewardship and AMR.

Overall, the panel echoed thoughts that were extracted from the interviewees. They thought that current care pathways for infection would benefit from rule-in or rule-out tests. Rule-out tests are more important from an antibiotic stewardship perspective as tests which indicate that a patient does not have an infection would reduce unnecessary prescribing. Rule-out tests would also help to inform which patients can be safely discharged. Rule-in tests would be best placed in triage settings to indicate where tests or treatment needed to be escalated.

Tests in an ED need careful consideration. Often, there are 15–20 patients presenting to an ED per hour and as few as three nurses on duty. There should be consideration of the practicalities around performing the test, and the time required to carry them out where staff burden is high. Tests should be simple and quick to give an indication of how sick a patient is (or how sick they may get), in the early stages of presentation.

Clinical judgement is something that may at times override diagnostic test results, with clinicians often trusting judgement and expertise to a greater extent.

There are multiple points on the severe infection pathway where a new test would be useful. Its placement would be dependent on the type of test and on the type of patients being assessed. It is important to think about whether a particular test can address a broad demographic of patients, or whether different tests for different groups of people are required.

The following test characteristics were highlighted as important:

- A rapid test to allow for narrow rather than broad-spectrum antibiotic prescription for the sickest patients.
- For less severely ill patients, a test that would indicate whether the patient has an infection or not (yes/no).
- A test that can provide risk stratification and predict critical care admission, death and/or need for organ support.
- Ability of a test to distinguish viral from bacterial infection (with the caveat that it is possible for viral and bacterial pathogens to co-exist).
- A molecular test combined with assessment of host response is desirable.
- A POC test would be useful if there is room in the ED.
- The size of the machine is an important factor as there is limited space in EDs.
- The test should be located in an appropriate place so that it is safe and accessible to a range of staff.
- The test should have the capability to link up with EPRs.

Barriers and facilitators to test adoption were an important part of the panel discussion. The main feature that would support the adoption of a new diagnostic test for infection was if it would enhance the potential to discharge patients confidently and safely. This would provide patient benefit, benefit to the healthcare system and potential economic benefits.

There should be reasonable capacity in terms of space and staffing level in a department to host and perform tests. When introducing a new test, thought should be given as to whether there is sufficient staff capacity to perform and interpret additional tests, or tests should save time in the long run, for example by expediting results that would usually take time e.g., laboratory tests. These are key considerations in a busy triage setting.

It was shared that tests which are consultant-only access present a barrier to implementation in the respect that only a limited number of staff can use them. These tests would also be problematic in terms of generating “buy-in” from staff who are not authorised or qualified to perform or interpret the tests. Any new test should be accessible to all grades of healthcare staff who may be treating patients with potentially serious infections.

On the matter of staff buy-in, panel members commented that clinicians are often not at the forefront of decisions regarding the acquisition of new technology. The outcome of this is the kit that resides in the ED is occasionally not fit for purpose. Generally, it is at the hospital management level, procurement and commissioning that the approval of new tests takes place. Clinicians feel that they have less control now over the equipment acquired than they used to and much of the new kit procured is unsuitable. Ensuring that new tests are tried and tested or are accepted by clinicians and ED staff is important in ensuring acceptance and uptake.

The clinicians intimated that cost is an important consideration for test adoption although clinicians are not motivated by cost per se but by patient care as a whole. A good business case is required to implement new tests in an emergency setting. Thorough health economics assessment plays a significant role in the determination of which diagnostic tests to adopt.

The discussion ended with a fitting thought: that small studies in isolation do not drive national change regarding new test adoption or adoption of new standards of care and that there is a clear need for a large-scale platform trial to identify the most suitable test(s) for serious infection.

## Discussion

The aim of this work in developing the PROTECT platform was to elicit information from clinical experts on the diagnosis, treatment and management of patients at risk of severe infection. Key objectives were to describe the current care pathway including treatment and management for those with suspected infection, to identify appropriate care pathway entry points for new diagnostic tests which impart maximum clinical and cost-effectiveness and efficiency, and the identification of barriers and facilitators to introduction of new diagnostic tests for infection. These topics were explored with an underlying focus on antibiotic optimisation and AMR. Other priorities were to refine the project remit and inclusion/exclusion criteria for the platform study and to elicit expert opinion on the evaluation of diagnostic tests in the ED.

The aims were met via the collection of qualitative data in three contexts:

(i)  Small-group discussions to refine the PROTECT project remit and define the inclusion/exclusion criteria.

(ii)  Interviews with healthcare experts to ascertain current practice and clinical needs for diagnosing infection.

(iii)  A panel discussion with experts presenting the ED perspective on diagnostic testing for infection.

The small-group discussions, guided by prompts, facilitated definition of the project remit, inclusion and exclusion criteria for the study.

During the research interviews, healthcare experts expressed that a rapid diagnostic test to identify patients at risk of deteriorating from serious bacterial infection was a clinical need. Tests which assist with the stratification of patients into high- and low-risk groups so that treatment could be appropriately targeted, whilst also avoiding unnecessary or inappropriate prescribing of broad-spectrum antibiotics, would also be welcomed. Tests would be adjuncts to the standard-of-care tests that are currently performed. A new diagnostic test for infection would accompany clinical experience and judgement.

Participants spoke particularly about how new diagnostic tests could facilitate the management of “amber” patients for whom it was difficult to assess risk meaning that it was often unclear whether they could be safely discharged or had the potential to become seriously ill. In current practice, these patients are likely to be observed or treated “just in case”. Participants provided suggestions as to how a new test would definitively rule in or rule out the possibility of serious deterioration for these patients and what parameters would be used.

Participants were asked where a new diagnostic test would best fit within the current care pathway for infection. A large proportion of interviewees believed that earlier in the pathway was better. Participants were most interested in a test that could quickly rule out participants at triage who were not seriously ill or had less potential to become seriously ill and could therefore be discharged safely. Secondary to this was a test that would rule in patients with a serious infection who might otherwise be missed by traditional screening and diagnostic tests. These tests might facilitate decision-making for the “amber” group where there is clinical uncertainty over level of risk. There was also considered to be clinical need for a test which could distinguish between a bacterial or viral infection. This would effectively rule out antibiotic treatment for those with a viral infection in alignment with antimicrobial stewardship.

Clinicians were less interested in what was referred to as a “traditional diagnostic test”; for a rapid test, it was deemed more useful to be able to stratify patients by risk than indicate what the infective organism was. Priority was managing risk and safely treating patients. Stratifying patients into high- and low-risk groups would allow busy clinicians in EDs to focus on patients who were most at risk. Streamlining the classification of patients could alleviate pressure on busy EDs or assessment suites by freeing up bed spaces or expediting patient flow to the most appropriate parts of the care pathway.

Participants expressed that the test would need to be highly accurate (sensitive) in order for them to confidently discharge patients who were considered low risk. Further discussion may be needed regarding the level of risk that clinicians, and patients, are ready to accept in such a rule-out test. Participants suggested that patients are often willing to accept a higher level of risk than clinicians if the alternative is lots of tests in hospital. Work with patients, potentially through patient engagement groups is necessary to understand more about the patient perspective around diagnostic testing for infection. Generally, the ideal output of a new test would be a definitive binary result (yes/no regarding infection status) which did not require further interpretation.

A further potential use case presented by participants was for a test that allowed monitoring and measurements to be taken over time in order to assess the patients’ response to treatment. However, this would need to be faster or more efficient than tests currently available (for example CRP) otherwise the new test would not add benefit for the patient or to the pathway.

Overall, in terms of AMR, participants stated that stewardship was less of a priority in practice than risk to patients or preventable harm. Improved antibiotic stewardship that occurred as a result of the introduction of a new test would likely be incidental rather than an active priority for clinicians. The introduction of a new rapid infection diagnosis test to distinguish between patients who are infected and those who are not, would naturally reduce antibiotic prescribing in the first instance.

All participants suggested that there are challenges in introducing a new test or technology into the NHS. The consensus was that the main barrier to implementing a new test was resistance to change and behaviour change. This might be due to the perceived additional burden of performing extra tests. It might also be cultural resistance and a preference to do things the way they have always been done. Robust and high-quality evidence would be needed to convince staff that new tests are valid, accurate and of benefit. Overcoming cultural bias might require top-down and bottom-up endeavour with those responsible for test procurement and implementation championing the test and clinicians who would be using the test feeling confident about (or generating evidence of) the efficacy and efficiency of the test.

Overall, participants indicated that for a new test to be implemented successfully, it needs to benefit patients, clinicians, the care pathway and the NHS.

There was a large amount of consensus between the panel discussion and the views expressed during the interviews. The combined evidence allows developers to understand from a clinical perspective what tests are needed in an ED setting and what characteristics these should have.

### Study limitations

There were some limitations of this study. Although we reached what appeared to be evidence saturation, or consensus between participants, the number and range of stakeholders engaged in the interviews (n = 8) may not be adequately representative of all healthcare workers who interact with patients at risk of serious infection in primary and secondary care settings. The NMIC research group attempted to mitigate bias by ensuring a balance of expert specialties (acute adult specialists, paediatric acute specialists, a paramedic, a critical care nurse and an intensive care and acute specialist). Geographically, they were spread across the breadth of the UK. As the exact tests to be included in the PROTECT trial are not yet known, much of the discussion with participants was speculative, although the participants were able to set out criteria for how an ideal test should perform and what characteristics and specifications it should have. Further work could determine exactly how a new test could be implemented in order to offer the most benefit to patients and to clinical staff and systems.

## Conclusions

Participants were supportive of introducing a new test to improve the diagnostic and treatment pathway for patients with suspected serious infection whilst simultaneously improving antibiotic stewardship. It was felt that all tests should be used in combination with clinical judgement and, to be worthwhile, would need to improve the current care pathway in a way that benefitted patients, clinicians, and the health system. A rule-out test would be most advantageous as it would allow clinicians to identify and more confidently discharge patients who were unlikely to become seriously ill, safely reducing burden on busy EDs. A new test which was able to stratify “amber” or “indeterminate risk” patients as at high or low risk would be of significant benefit.

## Abbreviations list

**Table:** 

Abbreviation	Definition
AMR	Antimicrobial resistance
CPA	Care pathway analysis
CRP	C-reactive protein
ED	Emergency department
EPR	Electronic patient record
GP	General practitioner
HDU	High dependency unit
HTA	Health Technology Assessment
ICU	Intensive care unit
IT	Information technology
IV	Intravenous
MEWS	Modified Early Warning Score
NEWS	National Early Warning Score
NICE	National Institute for Health and Care Excellence
NMIC	Newcastle In Vitro Diagnostics Co-operative
PEWS	Paediatric Early Warning Score
POC	Point of care
PROTECT	Platform Randomised evaluation of clinical Outcomes using novel TEChnologies to optimise antimicrobial Therapy
qSOFA	Quick Sequential Organ Failure Assessment
RSV	Respiratory syncytial virus
RTI	Respiratory tract infection
SSTI	Skin and soft tissue infection
UTI	Urinary tract infection

## Ethical approval and consent

The study received ethical approval from the Newcastle University ethics committee on 25 ^th^ April 2023 (Ref: 30913/2023). Written or verbal audio-recorded informed consent was obtained from all participants prior to interviews taking place.

A participant information sheet and consent form were provided to participants via email prior to the interview date and participants were asked to return the signed consent form prior to the interview taking place. Participants who did not return the signed consent form were asked to consent verbally during the video call. In these instances, and in accordance with Newcastle University recommendations for consent, one methodologist would read out the statements from the consent form to the participant and a second methodologist would witness and record the verbal consent on the consent form.

## Figures and Tables

**Table:** 

Name	Affiliation
Alasdair Corfield	Royal College of Emergency Medicine Scotland, Glasgow and Clyde
Alasdair Gray	Royal College of Emergency Medicine NHS Lothian
Andrew Tabner	University Hospitals of Derby and Burton NHS Trust
Ashley (David) Price	NIHR Newcastle In Vitro Diagnostics Co-operative, Newcastle University
Bethany Shinkins	Warwick Medical School, Warwick University
Cat Atkin	Birmingham Acute Care Research Group, University of Birmingham
Cherry-Ann Waldron	Centre for Trials Research, Cardiff University
Clare Lendrem	NIHR Newcastle In Vitro Diagnostics Co-operative, Newcastle University
Dan Lasserson	Institute of Applied Health Research, Warwick University
David Gillespie	Centre for Trials Research, Cardiff University
Edd Carlton	Royal College of Emergency Medicine
Emma Thomas-Jones	Centre for Trials Research, Cardiff University
Enitan Carrol	Alderhey to Alder Hey Childrens NHS Foundation Trust/Liverpool University
Graham Prestwich	NIHR PPI
Gurdeep Sagoo	NIHR Newcastle In Vitro Diagnostics Cooperative, Newcastle University
James Wason	Population Health Sciences Institute, Newcastle University
Jane Freeman	NHS England/University of Leeds / Leeds Teaching Hospital Trust
Jane O'Hara	THIS Institute (The Healthcare Improvement Studies Institute), University of Cambridge
Joanne Euden	Centre for Trials Research, Cardiff University
Jonathan Sandoe	School of Medicine, University of Leeds
Jordan Evans	Cardiff and Vale University Health Board
Julie Carman	NIHR PPI
Liza Keating	Royal Berkshire Hospital
Luke Vale	London School of Hygiene & Tropical Medicine
Mandy Townsend	Health Innovation North West Coast
Margaret Ogden	NIHR PPI
Martina Svobodova	Centre for Trials Research, Cardiff University
Neil French	Liverpool University Hospitals NHS Foundation Trust/University of Liverpool
Nicola Howe	NIHR Newcastle In Vitro Diagnostics Co-operative, Newcastle University
Paul Dark	Faculty of Biology, Medicine and Health, University of Manchester
Philip Pallmann	Centre for Trials Research, Cardiff University
Philip Howard	NHS England/Leeds Teaching Hospitals NHS Trust
Raasti Naseem	Health Innovation North East and North Cumbria
Richard Body	University of Manchester/ Manchester University NHS Foundation Trust
Sara Pretorius	NIHR Newcastle In Vitro Diagnostics Cooperative, Newcastle University
Sarah Harle-Jones	NIHR PPI
Sarah Rawlinson	Centre for Trials Research, Cardiff University
Shalini Ahuja	Centre for Implementation Science, King’s College London
Shrouk Messahel	Alderhey to Alder Hey Childrens NHS Foundation Trust
Stacy Todd	Liverpool University Hospitals NHS Foundation Trust
Stephen Lynn	Health Innovation North East and North Cumbria
Steve Bell	North West Ambulance Service
Tom Waterfield	Wellcome Wolfson Institute for Experimental Medicine, Queen's University Belfast/‎Paediatric Emergency Research UK and Ireland

## Data Availability

Due to the qualitative nature of the data collected unrestricted data sharing is not available. Doing so could breach participant confidentiality. Furthermore, participants did not provide explicit consent for their data to be shared beyond the research team involved in this study. A reasonable request including the requestees qualifications, affiliations and proposed secondary use of data should be sent to the corresponding author. Requests will be considered on an individual basis and if granted an anonymised data set will be provided. Newcastle University: Extended data for ‘Mapping decision-making pathways: Determination of intervention entry points for diagnostic tests in suspected serious infection’, https://doi.org/10.25405/data.ncl.25577487 ^16^. This project contains the following extended data: Data file 1. Interview schedule used for the semi structured interviews. Data file 2. Questions and prompts used during the brainstorming session of the stakeholder meeting. Data are available under the terms of the Creative Commons Zero "No rights reserved" data waiver (CC0 1.0 Public domain dedication).
