# Supplementary material for: Mapping decision-making pathways: Determination of intervention entry points for diagnostic tests in suspected serious infection
Source: NIHR Open Res. 2025 Jan 17;4:35. Originally published 2024 Jun 7. [Version 2] doi: 10.3310/nihropenres.13568.2 (PMC11754954; doi:10.3310/nihropenres.13568.2)
Supplement: Peer review history

# Reviewer report: Reviewer response for version 2

**DOI:** 10.3310/nihropenres.15085.r34496

**Published:** 2025-01-22

**Authors:** Yasaman Fatemi

**Affiliations:** 1 Pediatric Infectious Diseases, University of Washington School of medicine, Seattle, Washington, USA

No additional comments.

Is the work clearly and accurately presented and does it cite the current literature?

Yes

If applicable, is the statistical analysis and its interpretation appropriate?

Not applicable

Are all the source data underlying the results available to ensure full reproducibility?

No source data required

Is the study design appropriate and is the work technically sound?

Yes

Are the conclusions drawn adequately supported by the results?

Yes

Are sufficient details of methods and analysis provided to allow replication by others?

Partly

Reviewer Expertise:

My area of expertise is diagnostic test stewardship, quality improvement methods, and qualitative methodology with a focus on clinical pathways.

I confirm that I have read this submission and believe that I have an appropriate level of expertise to confirm that it is of an acceptable scientific standard.

# Reviewer report: Reviewer response for version 1

**DOI:** 10.3310/nihropenres.14728.r32377

**Published:** 2024-09-16

**Authors:** Juan Pericas

**Affiliations:** 1 Vall d'Hebron University Hospital, Barcelona, Catalonia, Spain

Naseem and colleagues conducted a multi-phase qualitative study with various stakeholder groups involved in the field of serious infections to assess the priorities and main care pathway analysis aimed at establishing key aspects of clinical utility and cost-effectiveness of potential rapid diagnostic tests (RDTs) to be evaluated in a future platform trial. Authors made an in-depth analysis of multiple relevant aspects linked to the utility of such RDT by using two clinical use cases where they would be more largely utilised, i.e., emergency rooms and hospitalizations, which allowed to uncover fruitful nuances in their use which might have implications in how the master protocol for the potential future PT is designed. 

 I do not have any major comments. 

 As minor remarks: 

 1. Abstract. Please, revise the following sentence: "The platform will assess their comparative clinical and cost-effectiveness relative to current standard of care". Likely something is missing after "clinical". 

 2. Abstracts, results. While the first use case is specifically mentioned, the second use case is not. Please, either refer to it or remove the mention to the results concerning the first use case. 

 3. Abstract, conclusions. "Cost-effectiveness" missing. Moreover, these conclusions are quite vague and might apply to any field and type of diagnostic test. I suggest to try to be more specific. 

 4. Plain language summary. Although I agree in the whole with the message intended in the phrase "There is no reliable rapid test which can confirm bacterial

 infection, and current laboratory tests take too long to give results", this might be not accurate as a a non-nuanced generalisation (we might go into the rabbit hole defining realibility here, but test such as urinary antigens for S. pneumoniae, Legionella spp, etc). 

 5. Figure 1. Define the acronyms in the footnote. 

 6. Table 1. Please, explain with more detail how from 40 participants 49 responses were obtained. 

 7. Discussion. I think the readership would benefit from a more extensive discussion on the difficulties of tapping at the same time a public health issue such as AMR (collective appraisal, mid-term non-individual objectives) and a clinical challenge such as the antibiotic management of potential serious infections. This has strong implications on how cost-effectiveness is defined, for instance. Where public health experts and decision-makers involved in the discussions/interviews?

 8. Discussion. In relation with the prior point, I believe the readership will benefit from a short summary, perhaps in the first paragraph of the discussion, of how this study advances the knowledge in the field and provides insight that might serve to better design a PT in the field. Currently, there is a summary of the aims of the study, but I have not found a synthetic appraisal to the added value.

Is the work clearly and accurately presented and does it cite the current literature?

Yes

If applicable, is the statistical analysis and its interpretation appropriate?

Not applicable

Are all the source data underlying the results available to ensure full reproducibility?

Yes

Is the study design appropriate and is the work technically sound?

Yes

Are the conclusions drawn adequately supported by the results?

Yes

Are sufficient details of methods and analysis provided to allow replication by others?

Yes

Reviewer Expertise:

Infectious disease, public health

I confirm that I have read this submission and believe that I have an appropriate level of expertise to confirm that it is of an acceptable scientific standard.

# Reviewer report: Reviewer response for version 1

**DOI:** 10.3310/nihropenres.14728.r32133

**Published:** 2024-07-16

**Authors:** Yasaman Fatemi

**Affiliations:** 1 Pediatric Infectious Diseases, University of Washington School of medicine, Seattle, Washington, USA

This manuscript describes a multi-step qualitative approach to help design a platform trial to evaluate multiple diagnostic tests to improve testing for serious infections and improve antimicrobial stewardship in these contexts. This paper highlights the importance of practical methods to introduce new diagnostics tests and the need for broad stakeholder engagement. The breadth of stakeholder engagement including clinicians who are using these potential tests is a strength of this study.

 Comments:

 1) Methods - Stakeholder group discussions: How was the data from the small groups analyzed? Were these recorded/transcribed and then formally analyzed in themes or was this treated as informal discussion? It seems that the stakeholder discussion was mostly informal the way I am reading it. This can still be valuable but would be quite unstructured and difficult to analyze from a qualitative standpoint. there is more information about these discussions in the results section; however, typically it would be helpful to have this type of information about structure, number of participants, etc in the methods section.

 2) Methods - Semi-Structured Interviews: I would like to see more specificity about the semi-structured interview analysis method (e.g. was this content analysis?). It would also be helpful to know how the team arrived at the number of interviews. There are only 8 interviews -- was this predetermined or truly what was needed to reach thematic saturation?

 3) It would be helpful to summarize the themes of the semi-structured interviews before going into the detailed narrative. I found it hard to follow the big picture. This could also potentially be summarized in a figure instead. Defer this need to the editor.

Is the work clearly and accurately presented and does it cite the current literature?

Yes

If applicable, is the statistical analysis and its interpretation appropriate?

Not applicable

Are all the source data underlying the results available to ensure full reproducibility?

No source data required

Is the study design appropriate and is the work technically sound?

Yes

Are the conclusions drawn adequately supported by the results?

Yes

Are sufficient details of methods and analysis provided to allow replication by others?

Partly

Reviewer Expertise:

My area of expertise is diagnostic test stewardship, quality improvement methods, and qualitative methodology with a focus on clinical pathways.

I confirm that I have read this submission and believe that I have an appropriate level of expertise to confirm that it is of an acceptable scientific standard, however I have significant reservations, as outlined above.

# Response

**Published:** 2025-01-16

**Authors:** Nicola Howe

**Affiliations:** Faculty of Medical Sciences, Newcastle University, Newcastle upon Tyne, England, UK

This manuscript describes a multi-step qualitative approach to help design a platform trial to evaluate multiple diagnostic tests to improve testing for serious infections and improve antimicrobial stewardship in these contexts. This paper highlights the importance of practical methods to introduce new diagnostics tests and the need for broad stakeholder engagement. The breadth of stakeholder engagement including clinicians who are using these potential tests is a strength of this study.

**We thank the reviewer for their comments and the time taken to review our manuscript. We have responded to each comment and made amendments to the manuscript as applicable.**

 1) Methods - Stakeholder group discussions: How was the data from the small groups analyzed? Were these recorded/transcribed and then formally analyzed in themes or was this treated as informal discussion? It seems that the stakeholder discussion was mostly informal the way I am reading it. This can still be valuable but would be quite unstructured and difficult to analyze from a qualitative standpoint. there is more information about these discussions in the results section; however, typically it would be helpful to have this type of information about structure, number of participants, etc in the methods section.

**We have added more detail to the methods section regarding the collection and analysis of the data from the small group discussions.**

 2) Methods - Semi-Structured Interviews: I would like to see more specificity about the semi-structured interview analysis method (e.g. was this content analysis?). It would also be helpful to know how the team arrived at the number of interviews. There are only 8 interviews -- was this predetermined or truly what was needed to reach thematic saturation?

**We have added further detail regarding the analysis of the semi-structured interview data to the methods section.**

**We have also added further detail to the methods section regarding the number of participants we intended to interview.**

**We have also explained in the results section that we felt we had reached enough variation in interviewee roles and setting at 8 participants (combined with the relatively short timescale in which to identify and interview participants outlined in the methods).**

 3) It would be helpful to summarize the themes of the semi-structured interviews before going into the detailed narrative. I found it hard to follow the big picture. This could also potentially be summarized in a figure instead. Defer this need to the editor.

**We have amended the second paragraph of the semi-structured results to explain that the data is presented under the headings of the discussion guide: “(i) current procedure for diagnosing infection, (ii) uses and characteristics of tests which could potentially be added into the care pathway of patients with suspected infection (including clinical scenario, clinical setting, patient population and position in the care pathway) and (iii) implementation barriers and facilitators to adoption for tests which might be introduced into the care pathway for patients with suspected infection.”**
